# Ethanol Extract of* Securidaca longipedunculata* Induces Apoptosis in Brain Tumor (U87) Cells

**DOI:** 10.1155/2019/9826590

**Published:** 2019-02-26

**Authors:** Saidu I. Ngulde, Umar K. Sandabe, Roger Abounader, Thomas K. Dawson, Ying Zhang, Ibrahim Iliya, Isa M. Hussaini

**Affiliations:** ^1^Department of Veterinary Pharmacology and Toxicology, Faculty of Veterinary, University of Maiduguri, PMB 1069, Maiduguri, Borno State, Nigeria; ^2^Department of Veterinary Physiology and Biochemistry, Faculty of Veterinary, University of Maiduguri, PMB 1069, Maiduguri, Borno State, Nigeria; ^3^Departments of Microbiology, Immunology and Cancer Biology, University of Virginia, PO Box 800168, Charlottesville, VA 22908, USA; ^4^Chemistry Department, University of Virginia, PO Box 800168, Charlottesville, VA 22908, USA; ^5^Department of Pharmaceutical Chemistry, Faculty of Pharmacy, University of Maiduguri, PMB 1069, Maiduguri, Borno State, Nigeria; ^6^Department of Pharmacology and Toxicology, Faculty of Pharmacy, University of Maiduguri, PMB 1069, Maiduguri, Borno State, Nigeria

## Abstract

Incidence of cancer is estimated to be on the increase and current anticancer drugs are characterized by narrow margin of safety and side effects. There is the need to explore new drugs especially from plants since plants serve as major source of drugs.* Securidaca longipedunculata *Fresen plant is called the mother of all medicines in northern Nigeria and is used traditionally in the treatment of cancers by most traditional medicine practitioners in the region. This study is aimed at evaluating the anticancer activity of the plant extract using U87 brain tumor cell line. Ethanol extract of its root bark was prepared and fractionated by silica gel column chromatography.* In vitro* activity of the extract and fractions were assessed on the viability of U87 malignant brain tumor cell line by using hemacytometer, annexin V-PE and 7AAD flow cytometry and western blot detection of Poly-ADP-Ribose-Polymerase (PARP) cleavage. The results showed that the extract significantly (p<0.01) inhibited proliferation of U87 cell line with IC_50_ of 20.535 *μ*g/ml. Apoptosis was induced by the extract (41.53 ± 10.33%) and the polar fraction (47.3 ± 2.7%) via cleavage of PARP. It was concluded that the ethanol extract of* S. longipedunculata* root bark inhibited proliferation of U87 cell line and induced apoptosis by cleavage of PARP, thus supporting folkloric use of the plant in the management of cancers.

## 1. Introduction

Cancer is a disease characterized by uncontrolled cell proliferation in the body, leading to impaired functions and high mortality. In 2012, it was estimated that there were 14 million new cases and 8.2 million cancer related deaths and by the year 2050; this could increase to 27 million cases, 17 million deaths and 75 million persons living with cancer [1, 2].

Man has been using plants as source of drugs since time immemorial and today plants are still the major source of new drugs. Plants would still serve as potential avenue for the discovery and synthesis of new drugs in the near future or even beyond [3]. Drugs such as vincristine, vinblastine, vinorelbine, paclitaxel, docetaxel, etoposide, camptothecin, topotecan and irinotecan are plant-derived or semi-synthetically produced from the isolated analogs and are used clinically in the treatment of cancers. The use of these drugs is characterized by narrow margin of safety and side effects.

The plant* Securidaca longipedunculata *Fresen (violet tree) belonging to the family* Polygalaceae *is known as the mother of all medicines in Hausa language in Northern Nigeria. Its extract is used by Traditional Medicine Practitioners (TMPs) in the management of many ailments including cancer in African countries. It is the most frequently used plant in the management of cancer by TMPs in Northeastern Nigeria [4]. Research conducted on the extract demonstrated antiproliferative activities on Ehrlich ascites carcinoma* in vitro *and* in vivo*, decreasing angiogenesis and inducing DNA fragmentation [5]. In this report we demonstrated that the extract of* S. longipedunculata* root bark inhibited proliferation and induced apoptosis in U87 brain tumor cell line by cleaving Poly-ADP-Ribose-Polymerase (PARP).

## 2. Methods

### 2.1. Plant Collection and Identification

*Securidaca longipedunculata *was collected from Ngulde district in Borno State, Nigeria. It was confirmed and authenticated by Prof. S. S. Sanusi of the Department of Biological Science, and a voucher specimen (Vet212A2) was deposited at the Veterinary Pharmacology Laboratory, University of Maiduguri, Nigeria.

### 2.2. Extract Preparation

The root of the plant was collected, cleaned, and the bark removed, crushed and then air-dried at room temperature for one week. It was pulverized using a mortar and pestle and the ground herb was soaked overnight in petroleum ether at 1:5. The residue from defatted samples was extracted in 95% ethanol at 1:5 for 24h. The sample was filtered using Whatman filter paper No. 1 and evaporated to dryness under reduced pressure using a rotary evaporator.

### 2.3. Fractionation of Extract

The extract was fractionated on silica gel (60-230 mesh size) column chromatography using an increasing gradient of methanol in chloroform up to 100%. Every 15 ml of the eluate was collected in a test tube and those with similar thin layer chromatography (TLC) composition were combined to obtain 7 fractions labeled AI - AVII. Thin layer chromatography was developed in a glass tank, dried and visualized using UV light 254 wavelength and Seebach's dip. Each fraction was evaporated to dryness under reduced pressure using a rotary evaporator and stored at 4°C.

### 2.4. Cell Line and Cell Culture Conditions

Malignant brain tumor cell line (U87) was obtained from Dr. Abounader's laboratory, University of Virginia Health System, Charlottesville, USA. The cell line was cultured in minimal essential medium-*α* (MEM- *α*) supplemented with 10% fetal bovine serum (FBS), 1% sodium pyruvate, 2% sodium bicarbonate, 1% non-essential amino acid and 1% penicillin/streptomycin. The culture condition was 37°C and 5 % CO_2_.

### 2.5. Proliferation Assay

The U87 cell line was seeded in 6-well plates at 40,000 cells (or 20,000 if 12-well plates) in triplicates using the standard media as described by Guessous* et al.,* [6]. Cells were incubated overnight before treated with the extract/fraction at concentrations 3, 10, 30, and 100 *μ*g/ml and then control wells. After 12-48 h of incubation, the cells were trypsinized, harvested and counted using hemocytometer. Median inhibitory concentration (IC_50_) was estimated from percentage inhibition-log concentration curve using Microsoft excel version 14.1.0.

### 2.6. Annexin V-PE and 7AAD Flow Cytometry

The U87 cell line was seeded at 50,000 cells in triplicates using the standard growth media in 6 well plates. Cells were incubated overnight before treated with extract at 10 *μ*g/ml for 48 h. The cells were harvested and stained with Annexin V and 7AAD (7-aminoactinomycin D) as described by Li* et al.,* [7]. After 15 minutes of incubation, flow cytometric analysis was conducted using FACsan and apoptotic fractions were determined.

### 2.7. Western Blotting

The U87 cell line was seeded in 10 mm^3^ petri dishes at 200,000 cells per dish in 10 ml media. After 24 h incubation, cells were treated with the extract by replacing the media with the one containing the extract/extract fraction (10 *μ*g/ml). It was incubated for 48 h including control dishes. Cells were harvested and protein contents extracted using the RIPA buffer (1 % Igepal, 0.5 % sodium deoxycholate, 0.1 % SDS in PBS). Extracted proteins were kept at -20°C for further use. Protein concentration was determined using Coomassie plus reagent (Bradford assay) and absorbance was taken using the ELISA reader.

The western blot was performed as described by Zhang* et al.,* [8]. Equal amounts of proteins were loaded and separated in a Sodium dodecyl sulfate-polyacrylamide gel electrophoresis (SDS-PAGE) (Invitrogen, Carlsbad, CA) at 140V (initial setting at 60V for 20 minutes) for 1.5h at room temperature and then transferred to a nitrocellulose membrane at 35V for 1.75h at 4°C. The membrane were rinsed in TBST and then blocked with 5% skimmed milk in TBST for 1 h at room temperature. The membrane was incubated with the primary antibodies (anti-PARP, anti-cleaved PARP and anti-actin) in fresh 5% skimmed milk TBST at 4°C overnight on slow shaker. The antibody-bound membranes were washed 3 times in TBST each for 10 minutes. They were then treated with the specific secondary antibody in 5% skimmed milk TBST and incubated for 1h at room temperature on slow shaker followed by washing 3 times. The immunoreactive signals were detected with Super Signal Ultra chemiluminescent substrate (Thermo Scientific, Rockford, IL, USA).

### 2.8. Statistical Analysis

Treatments were performed in triplicate and data obtained were expressed as mean ± standard error of the mean (SEM) and analyzed statistically by one-way analysis of variance (ANOVA). Microsoft Excel (2011) for Mac and Graphpad Prism® version 4.00 for windows were used for data presentation and analyses. P <0.05 was considered significant.

## 3. Results

### 3.1. Effects of Securidaca longipedunculata Root Bark Ethanol Extract on the Proliferation of U87 Cell Line

**Figure 1:**
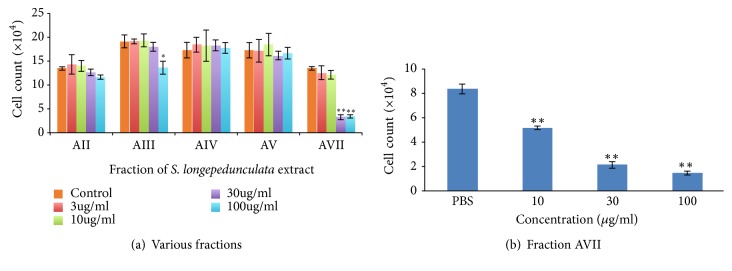
Effect of* S. longipedunculata* root bark ethanol extract on the proliferation of U87 cell line. The extract of* S. longipedunculata *was obtained using ethanol and fractions were obtained by column chromatography using increasing gradient of methanol in chloroform. The extract and fractions were used to treat U87 cell line at various concentrations for 48 h and cells harvested and counted by hemocytometer. Two fractions have antiproliferative activity (*∗∗* = p<0.01, *∗* = p<0.05) compared to the control (chart (a)), and the most polar fraction (AVII) has significant (*∗∗* = p<0.01) concentration-dependent anti-proliferative activity on U87 cell line with IC_50_ =20.535*μ*g/ml determined by Microsoft excel (chart (b)).

To identify the active fraction of the extract that is responsible for anti-proliferative activity, we performed column chromatography of the ethanol extract of* S. longipedunculata* root bark. As shown in Figure 1(a), the fraction AVII significantly (p<0.01) inhibited U87 proliferation at 30 and 100 *μ*g/ml. The other fractions had no significant (p>0.05) anti-proliferative activity. In Figure 1(b), the fraction AVII inhibited proliferation of U87 cell line significantly (p<0.01) at 10, 30 and 100 *μ*g/ml and the IC_50 _was found to be 20.535 *μ*g/ml.

### 3.2. Effect of Securidacalongipedunculata Extracts on Apoptosis

**Figure 2:**
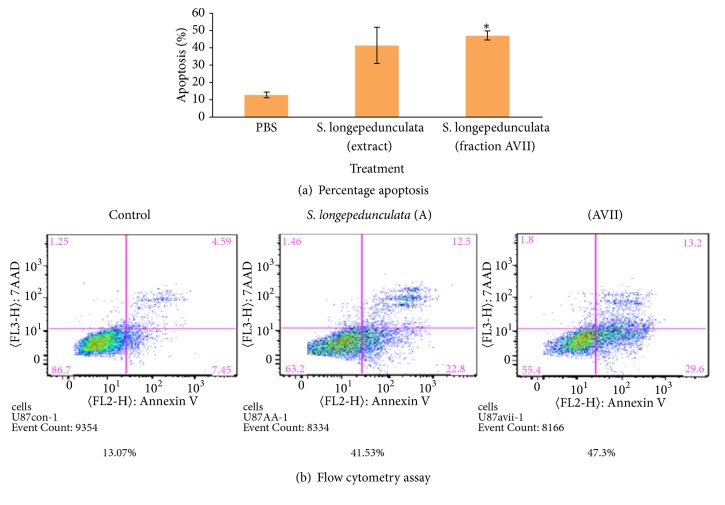
Effect of extracts of* S. longipedunculata *root bark ethanol extract and fractions on apoptosis in U87 cell line. U87 cell line was treated with the extract and most polar fraction of the extract of* S. longipedunculata *at 10 *μ*g/ml for 48 h. The cells were stained with Annexin V and 7AAD and then analyzed cytometrically using FACscan to determine the percentage apoptotic fractions. There was significant (*∗* =p<0.05) induction of apoptosis in treated wells compared to the control.

**Figure 3:**
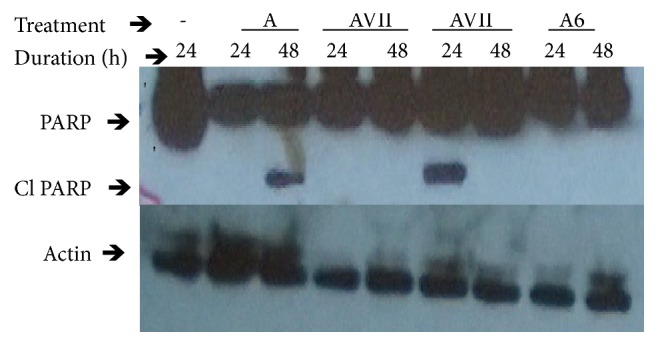
Effect of* S. longipedunculata *ethanol extract and fraction on cleavage of PARP protein in U87 cell line. U87 cell line was treated with the extract (A) and most polar fraction (AVII and A6 obtained from different batches fractionation) of the extract of* S. longipedunculata* at 10 *μ*g/ml for 24 h and 48 h.* Poly*-ADP-Ribose-Polymerase (PARP) and cleaved PARP (Cl) were determined by Western blot using specific antibodies. Cleaved PARP (Cl) is expressed in cells treated for 48 h with the extract and in cells treated with one of the fractions for 24 h.

One of the hallmarks of cancer is the evasion of apoptosis. To determine whether the crude extract of* S. longipedunculata* root bark and fraction AVII induce apoptosis, we performed Annexin V/7AAD flow cytometry and assessed for cleaved PARP (Poly-ADP-Ribose-Polymerase. Annexin V/7AAD flow cytometric analysis of the treated U87 cell line with the extract and fraction is presented in Figures 2(a) and 2(b). The extract and fraction showed induction of apoptosis of 41.53 ±10.33% and 47.3 ±2.7% respectively, and the latter is significantly (p<0.05) higher than the control (13.07±1.65%). The western blot analysis of U87 cell line after treatment with the extract and fraction is presented in Figure 3. There was induction of apoptosis as shown by the expression of cleaved PARP (Poly-ADP-Ribose-Polymerase) in the extract treated dish and in the dish treated with one of the batches of the fractions. Cleaved PARP was not expressed in the control dishes and dishes treated with the batches of the fractions that stayed long after fractionation. Cleaved PARP was expressed 24 h post treatment with the freshly extracted active fraction and 48 h post treatment with the extract.

## 4. Discussion

This research is on the activity of* S. longipedunculata* root bark ethanol extract on U87 brain tumor cell line.* Securidaca longipedunculata *is extensively used in African traditional medicine and is referred to as mother of all medicine in Northern Nigeria. It is the most frequently used plant by herbalist in the management of cancer [4]. Our result in this work supported the folkloric use as the ethanol extract inhibited proliferation of U87 cell line. The most polar fraction of the extract was responsible for the activity with IC_50_ of 20.535 *μ*g/ml. Antiproliferation may arise as result of the toxic effect of the plant leading to necrosis or may be due to programmed cell death. In order to study mechanism of antiproliferation, assessments of apoptosis were conducted using annexin V/7AAD flow cytometry and mechanisms of inducing apoptosis were performed by western blot detection of cleaved PARP. Flow cytometry showed there was significant (p<0.01) induction of apoptosis after treatment of U87 cell line with the extract and fraction. This was consistent with the results of antiproliferation as highest activity was observed in the polar fractions of* S. longipedunculata*.

Moreover, western blot analysis of PARP showed that there was cleavage of PARP in cells treated with the extract and the polar fraction of* S. longipedunculata* (Figure 3). PARP is a protein that binds specifically at DNA strand breaks. It also serves as substrate for certain caspases (caspases 3 and 9) and activated during early stages of apoptosis [9, 10]. It is an 115kDa protein that is activated by cleavage to produce two fragments, the activated PARP (85kDa) at the carboxy terminal domain from a 24kDa moiety at the amino terminal domain. It is a highly specific and sensitive marker for cells that are undergoing apoptosis [11]. Therefore it can be deduced that extracts of* S. longipedunculata* root bark especially the polar fractions induced apoptosis in U87 brain tumor cell line 24 h post treatment. As reported by Lawal* et al.* [5],* S. longipedunculata* extract 10 mg/kg increased the life span of tumor-bearing mice by reducing the viability of the tumor cells. A muchimangin isolated from the root of this plant recorded preferential cytotoxicity with PC_50_ value of 38.9 *μ*M in human pancreatic cancer PANC-1 cell line (Dibwe* et al.*, [12]. While there are reports on the activity of the extract of* S. longipedunculata* on cancer cells, there is no literature on the mechanism of inducing apoptosis. This work has revealed for the first time that apoptosis is induced by activation of PARP and the observed antiproliferations are due to apoptosis wholly or partly. This may be a promising avenue for the development of new anticancer drug since one of the hallmarks of cancer is evasion of apoptosis. Thus, this has defined our future direction in the search of new chemotherapeutic agents for the treatment of cancer. There is the need to isolate and identify the polar compounds in the extract responsible for induction of apoptosis. The use of other cancer cell lines to validate what is obtainable in this research is necessary.* In vivo *anticancer study using this plant can provide better insight into its activity in biological systems as used by TMPs. The distribution of polar compounds into the brain is restricted in intact blood-brain-barrier and whether there are non-polar compounds serving as adjuvant that are important in distribution or not is yet to be ascertained. The adjuvant may be from the same plant or another plant as TMPs often use a recipe of plants in the management of cancers.

## Data Availability

The data are included within the supplementary information file.
